# Production of a 135-residue long N-truncated human keratinocyte growth factor 1 in *Escherichia coli*

**DOI:** 10.1186/s12934-023-02097-z

**Published:** 2023-05-11

**Authors:** Young Su Kim, Hye-Jeong Lee, Gabriella Aphrodita Handoko, Jaehui Kim, Seong-Bo Kim, Minho Won, Jung-Ho Park, Jungoh Ahn

**Affiliations:** 1 Biotechnology Process Engineering Center, KRIBB, Cheongju, 20736 Republic of Korea; 2 Bio-Evaluation Center, KRIBB, Cheongju, 20736 Republic of Korea; 3 Department of Biosystems and Bioengineering, KRIBB School of Biotechnology, Korea University of Science and Technology (UST), 217 Gajeong-ro, Yuseong-gu, Daejeon, Korea; 4 Bio-Living Engineering Major, Global Leaders College, Yonsei University, 50 Yonsei-ro, Shinchon-dong, Seodaemun-gu, Seoul, 03722 Korea

**Keywords:** Palifermin, KGF-1, *Escherichia coli*, N-terminal truncation, Recombinant protein production

## Abstract

**Background:**

Palifermin (trade name Kepivance®) is an amino-terminally truncated recombinant human keratinocyte growth factor 1 (KGF-1) with 140 residues that has been produced using *Escherichia coli* to prevent and treat oral mucositis following radiation or chemotherapy. In this study, an amino-terminally shortened KGF-1 variant with 135 residues was produced and purified in *E. coli*, and its cell proliferation activity was evaluated.

**Results:**

We expressed soluble KGF-1 fused to thioredoxin (TRX) in the cytoplasmic fraction of *E. coli* to improve its production yield. However, three N-truncated forms (KGF-1 with 140, 138, and 135 residues) were observed after the removal of the TRX protein from the fusion form by cleavage of the human enterokinase light chain C112S (hEK_L_ C112S). The shortest KGF-1 variant, with 135 residues, was expressed by fusion with TRX via the hEK_L_ cleavage site in *E. coli* and purified at high purity (> 99%). Circular dichroism spectroscopy shows that purified KGF-1_135_ had a structure similar to that of the KGF-1_140_ as a random coiled form, and MCF-7 cell proliferation assays demonstrate its biological activity.

**Conclusions:**

We identified variations in N-terminus-truncated KGF-1 and selected the most stable form. Furthermore, by a simple two-step purification, highly purified KGF-1_135_ was obtained that showed biological activity. These results demonstrate that KGF-1_135_ may be considered an alternative protein to KGF-1.

**Supplementary Information:**

The online version contains supplementary material available at 10.1186/s12934-023-02097-z.

## Background

Keratinocyte growth factor-1 (KGF-1) is a superfamily of the fibroblast growth factor (FGF) with heparin-binding characteristics that promotes the growth of epithelial cell [1–3]. This growth factor, produced by the mesenchymal cell, interacts with FGF receptor 2 (FGF receptor-2111b) specifically expressed in stromal fibroblasts from epithelial tissues [4].

The characteristics of KGF-1 that promote cell proliferation and differentiation play an important role in repairing the epithelium in various tissues and organs in the early stages of wound healing [5, 6]. Owing to these restorative functions, it has been developed as an oral drug (palifermin) for mucositis treatment by Amgen [7]. Furthermore, many studies have suggested that KGF-1 stimulates the growth of hair follicles and represses hair growth during the telogen phase [8–11].

The original KGF-1 cNDA, encoding 194 residues, is isolated from a human embryonic lung fibroblast cell line (M426). It contains 31 residues of signal peptide and five N- and O- glycosylation sites [3, 5]. Two disulfide bonds (at the positions between 1 and 15 and between 102 and 106) and a free sulfhydryl Cys at position 40 are present in the mature form [3]. Thus far, KGF-1 has been produced using various hosts, such as Chinese hamster ovary (CHO) cells [12], plants [13], silkworms [14], *Pichia pastoris* [5], and *Escherichia coli* [7, 15], and with various modifications, including the expression of a different construct and fusion protein with glutathione-S-transferase (GST). However, when KGF-1 was produced in *E. coli*, a truncated form with 140 amino acids was observed. KGF-1_140_, also known as palifermin, is the most stable form of KGF-1 variants purified from *E. coli* [16, 17].

In this study, we demonstrate that KGF-1 fused with thioredoxin (TRX) reduced the degradation of KGF-1 and improved its production yield in the cytoplasm of *E. coli*. In addition, with the exception of KGF-1_140_, the amino-terminally shortened KGF-1_135_ was identified as the most stable and bioactive form.

## Results

### Amino-terminally truncated KGF-1

**Fig. 1:**
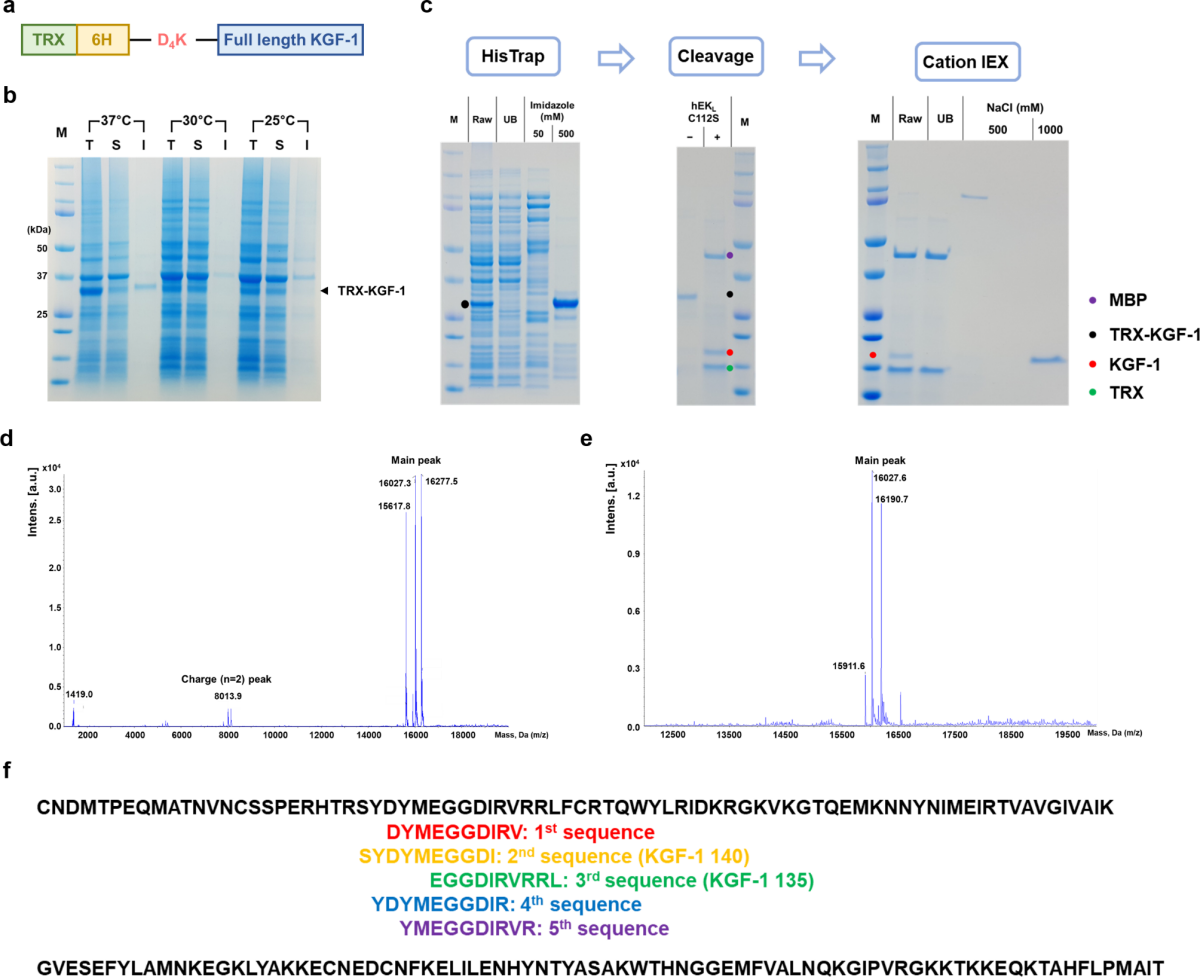
Expression and purification of full-length KGF-1 using *E. coli*. (**a**) Construction of KGF-1 fusion protein connected via the EK cleaved site (D_4_K). (**b**) Expression of the KGF-1 fusion protein in various temperatures. (**c**) Purification of KGF-1 with HisTrap and HiTrap SP. (**d, e**) LC-MS/MS analysis results of purified KGF-1. (**f**) N-terminal sequence data of purified KGF-1. T, total fraction; S, soluble fraction; I, insoluble fraction; M, protein marker; UB, unbound fraction (flow-through fraction); Black, target sequences; yellow, known as a highly stable sequence; green, the smallest sequence

**Fig. 2:**
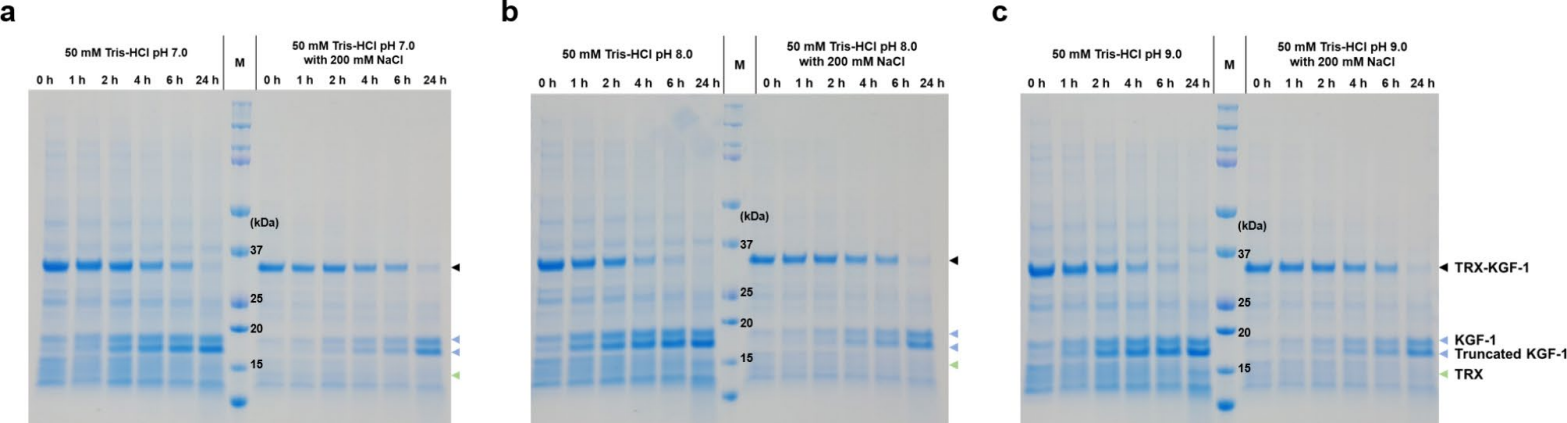
Cleavage reaction of TRX-6 H-KGF-1 fusion protein by hEKL C112S on various conditions. The cleavage reaction was conducted at (**a**) pH 7.0, (**b**) pH 8.0, and (**c**) pH 9.0 with or without 300 mM NaCl.

The expression vector for KGF-1 in *E. coli* was constructed as follows: the N-terminal of KGF-1 was fused to the solubility-enhancing fusion partner TRX by hexahistidine (6 H) and the enterokinase cleavage site (D_4_K) (Fig. 1a). The resulting fusion protein (TRX-6 H-D_4_K-KGF1) was expressed in a soluble form in *E. coli* BL21*(DE3*)/pET-30-TRX-6 H-D_4_K-KGF-1 at both 30 °C and 25 °C (Fig. 1b). The KGF-1 protein was purified by immobilized metal affinity chromatography (IMAC), TRX was removed by cleavage by hEK_L_ C112S, and cationic chromatography was performed (Fig. 1c). However, liquid chromatography-mass spectroscopy (LC-MS/MS) analysis and N-terminal sequencing demonstrated the presence of three amino-terminally truncated KGF-1 protein variants (16,269.3, 16,019.2, and 15,610.0 Da) (Fig. 1d, , Table S1). To identify whether the fragmentation of KGF-1 may be caused by non-specific cleavage owing to buffer conditions, we tested various cleavage reactions in different pH (pH 7.0, 8.0 and 9.0) and salt concentrations (0 and 200mM NaCl). However, N-truncated KGF-1 fragments were detected when cleavage reaction began under all conditions (Fig. 2).

### Production of a stable form of KGF-1_135_ in E. coli

**Fig. 3:**
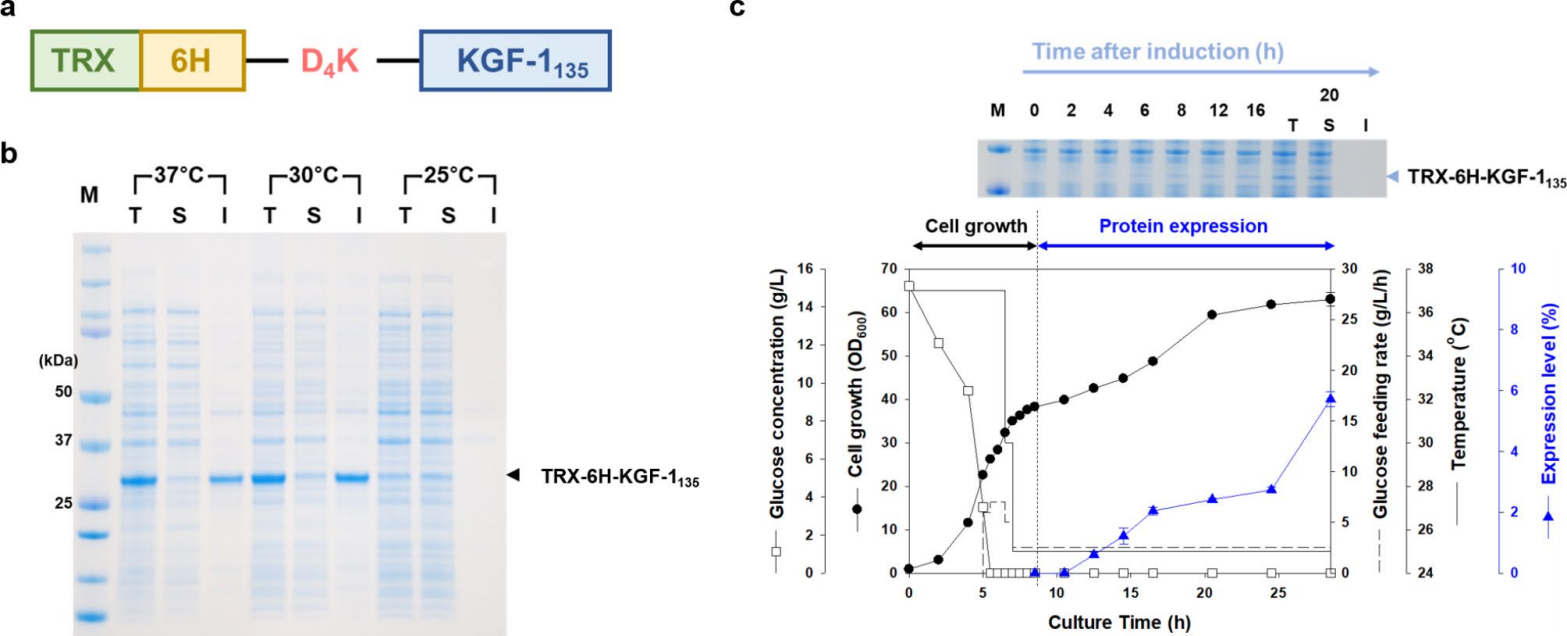
Construction of fusion proteins and expression tests in shake flask culture and fed-batch fermentation. (**a**) KGF-1_135_ was fused with TRX via the D_4_K. (**b**) The KGF-1 135 fusion protein was expressed in flask culture at 25, 30, and 37 °C. (**c**) Fed-batch fermentation and expression profiles of TRX-6 H-KGF-1_135_. T, total fraction; S, soluble fraction; I, insoluble fraction; M, protein marker; Inlet image of SDS-PAGE indicates the expression levels of fusion proteins

To produce single form of KGF-1, the expression vector for KGF-1_135_ in *E. coli* was constructed by fusion with TRX-6 H-D_4_K (Fig. 3a). The fusion protein (TRX-6 H-D_4_K-KGF1_135_) was expressed in the soluble form at only 25 °C in flask cultivation, unlike the full-length KGF-1 (Fig. 3b). For large-scale production of KGF-1_135_, 5 L fed-batch fermentation of KGF-1_135_ was attempted and optimized (Fig. 3c). After the initial glucose was completely consumed, glucose was fed into the fermenter at an appropriate rate to maintain the growth and glucose-limited conditions and improve the volumetric production yield. When the cells reached 35 OD_600_, the temperature was decreased to 25 °C, followed by the addition of lactose (final concentration: 15 g/L). The fusion protein (TRX-6 H-D_4_K-KGF-1_135_) was expressed after 4 h of induction, and its expression levels increased slowly up to 8 h. The final expression levels of TRX-6 H-D_4_K-KGF-1_135_ reached 5.7%, with 100% solubility (Table 1). The final cell concentration was 22.1 g_DCW_/L, and the volumetric yield of the fusion protein (TRX-6 H-D_4_K-KGF-1_135_) was approximately 0.6 g/L.

**Table 1:** Production of Trx-KGF-1_135_ using fed-batch fermentation

KGF	Endpoint OD_600_	Dry cell weight (g/L) (wet cell) (g)	Expression level (%)^a^	Fusion protein (tag-free protein) (g/L)	Solubility (%)
KGF-1_135_	63.0 ± 0.0	22.1 (185.6)	5.7 ± 0.2	0.6 (0.4)	100

Data are shown as mean ± standard deviation from duplicate experiments

^a^Expression level and solubility were analyzed by densitometry using ImageJ with duplicate experiments

### Purification of KGF-1_135_

**Fig. 4:**
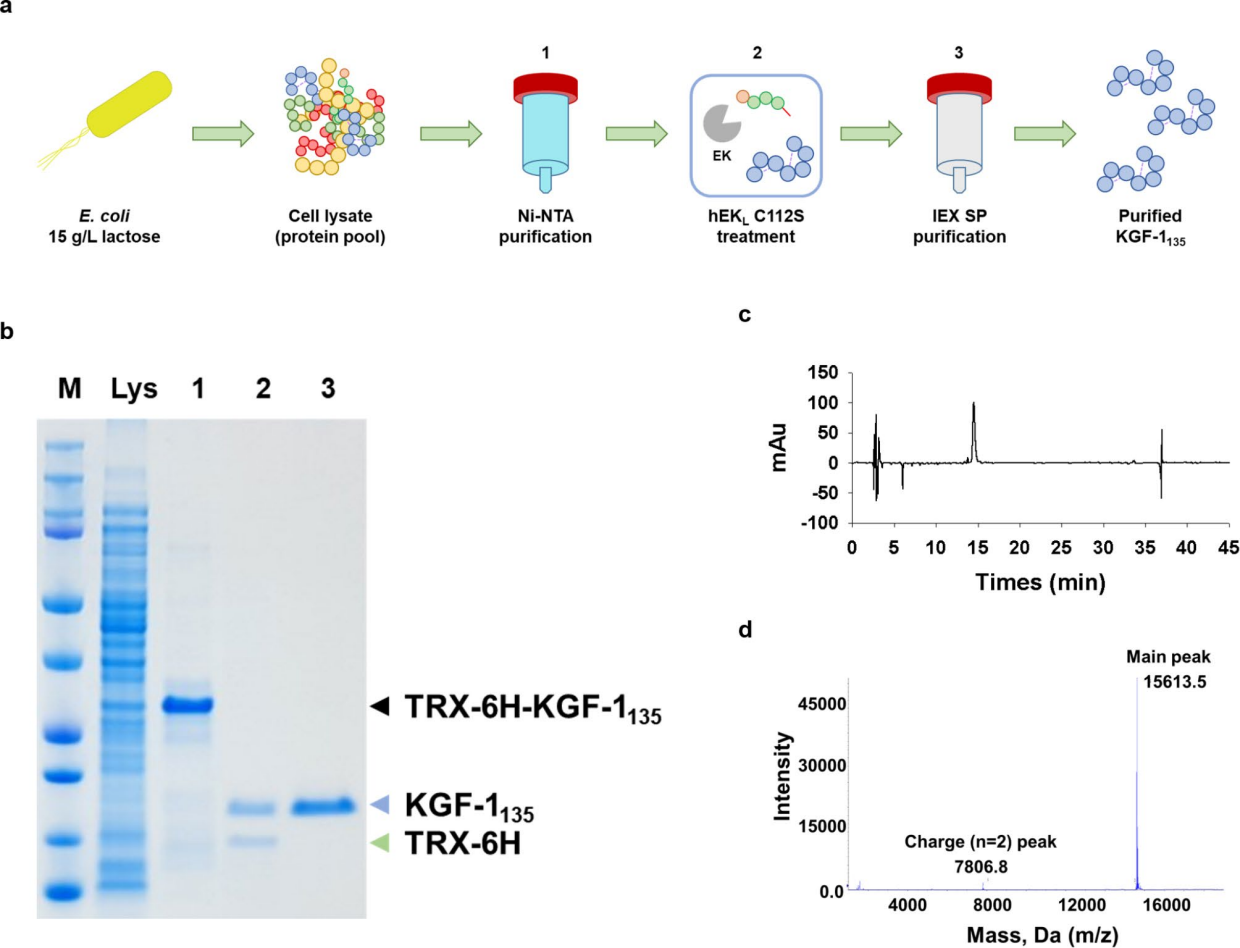
Purification and analysis of KGF-1 135. (**a**) Overall process of KGF-1_135_ purification. (**b**) SDS-PAGE of each purification step. (**c**) C18 RP-HPLC and (**d**) LC-MS (Q-TOF) analyses of purified KGF-1_135_. M, marker; Lys, supernatant after sonication; 1, HisTrap purification; 2, after EK treatment; 3, HiTrap SP purification (final product)

We developed a purification process to obtain KGF-1_135_ with high purity and recovery yield. As shown in Fig. 4a, the purification process of KGF-1_135_ consisted of IMAC, treatment with hEK_L_ C112S, and cation-exchange chromatography. Specifically, cell lysate containing TRX-6 H-D_4_K-KGF1_135_ was loaded onto the IMAC column (Fig. 4b, Lys), and the fusion protein was eluted from the column with the buffer containing 500 mM imidazole (Fig. 4b, lane 1). KGF1_135_, which was separated from TRX-6 H-D_4_K-KGF1_135_ by cleavage with hEK_L_ C112S (Fig. 4b, lane 2), was loaded onto the cation-exchange chromatography column for the removal of impurities and the TRX tag, and then eluted with 500 mM NaCl (Fig. 4b, lane 3). The eluted KGF-1_135_ showed 99% purity using C_18_ reverse-phase high-performance liquid chromatography (RP-HPLC) (Fig. 4c). LC-MS/MS analysis and N-terminal sequencing demonstrated only one peak with 15,613.5 Da and EGGDIRVRRL as N-terminal sequence, respectively (Fig. 4d). These results indicate that our production strategy produced KGF-1_135_ as a single form without truncated variants. The purification processes are summarized in Table 2. The overall purification yield was high (53%).

**Table 2:** Purification of the KGF-1_135_ from the fusion protein expressed in *E. coli*

Purification step	Concentration (mg/mL)	Volume (mL)	Total protein (mg)	Fusion protein (tag-free protein) (mg)	Purity (%)^a^	Yield (%)^b^
Crude extract	2.7 ± 2.1	70.0	190.3 ± 3.2	12.3 ± 4.0 (10.5 ± 0.3)	6.4	100
HisTrap, 5 mL	0.3 ± 0.0	60.0	19.2 ± 0.1	13.1 ± 0.2 (9.7 ± 0.3)	68.2	92.5
HiTrap SP, 5 mL	0.3 ± 0.0	20.0	5.6 ± 0.1	NA (5.6 ± 0.1)	100	53.0

^a^Purity was analyzed by densitometry using ImageJ and C18 RP HPLC.

^b^Yield was calculated by dividing the tag-free protein of each purification product by the tag-free protein in the crude extract

### Circular dichroism (CD) spectroscopy analysis of KGF-1_135_

**Fig. 5:**
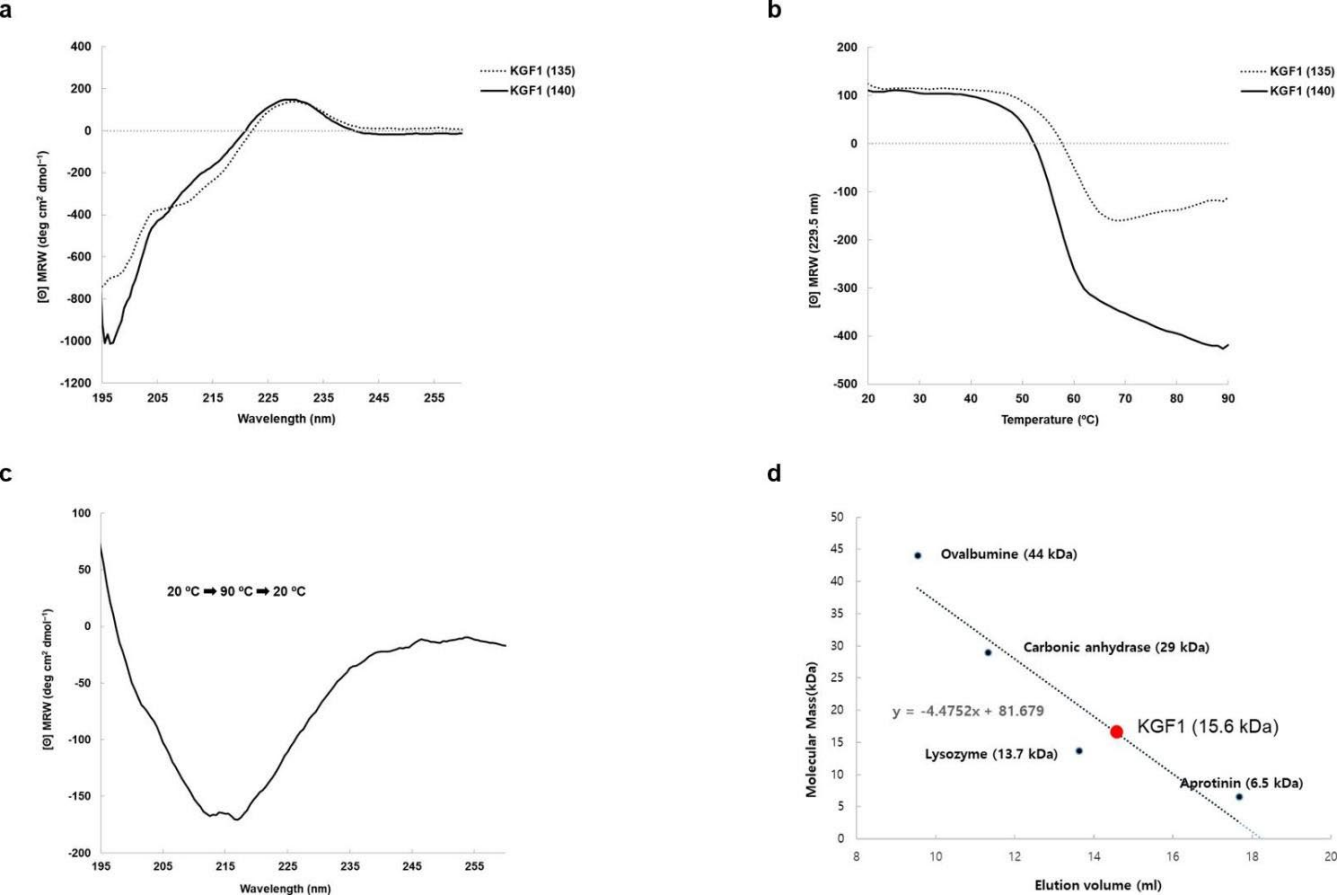
Analysis of (**a**) secondary structure and (**b**) thermal stability of purified KGF-1_135_ and KGF-1_140_ and (**c**) secondary structure of recovered KGF-1_135_ after thermal unfolding. (**d**) Gel filtration result of purified KGF-1_135_.

CD spectroscopy was used to characterize the secondary structure of the purified KGF-1_135_. The analysis showed a maximum and minimum mean residue ellipticity at 230 and 195 nm, respectively (Fig. 5a). This pattern indicates that the purified KGF-1_135_ had a typical random coiled structure and was almost the same as the reported CD spectrum of KGF-1_140_ [18]. Furthermore, it showed secondary structure similar to that of KGF-1_140_ which produced and refolded in *E. coli* (Fig. 5a). In addition, we evaluated the thermal stability of KGF-1_135_ at 20 to 90 °C at 229.5 nm (Fig. 5b). The folding structure was maintained up to approximately 45 °C, and the purified KGF-1_135_ was perfectly unfolded at 65 °C. These results indicated that the thermal stability of KGF-1_135_ was more stable than that of KGF-1_140_. To confirm whether the secondary structure of KGF-1_135_ is reversible after denaturation by heat, the temperature of KGF-1_135_ was raised to 90 °C and was decreased to 20 °C. The CD spectrometer results showed that thermal denaturation was irreversible (Fig. 5c). In addition, gel filtration analysis indicated that the purified KGF-1_135_ was present in monomeric form (Fig. 5d). This result shows that the thermal stability of KGF-1_135_ is within the normal range for cytoplasmic proteins [19].

### Cell proliferation assay of KGF-1_135_

**Fig. 6:**
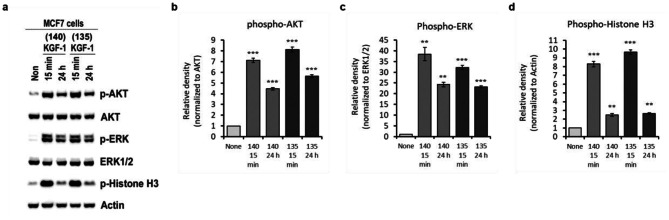
Cell signaling analysis of purified KGF-1_135_ and KGF-1_140_ related to the proliferation of MCF-7 cells. (**a**) Immunoblot analysis of proliferation related cell signaling. Quantitative evaluation of (**b**) phosphorylated AKT normalized by AKT, (**c**) phosphorylated ERK normalized by EKR1/2, and (**d**) phosphorylated Histone H3 normalized by actin. MCF7 cells were serum-starved for 24 h and then treated with KGF-1 for indicated time. Whole cell lysates of each sample were subjected to immunoblotting with the indicated antibodies. ^*^ indicated P < 0.05, ^**^ indicated P < 0.01, and ^***^ indicated P < 0.001

The biological activity of purified KGF-1_135_ was evaluated by analyzing the cellular signals after treatment of MCF-7 cells with KGF-1_135_ and KGF-1_140_. KGF-1 promotes proliferation via the mitogen-activated protein kinase (MAPK) pathway and PI3K/AKT signaling [20, 21]. In addition, generally, histone H3 is used to proliferation marker [22]. The KGF-1 induced cellular signaling (AKT, Extracellular signal-regulated kinase 1/2 (ERK) phosphorylation) is highly induced in 15 min and then decreased [23–25]. Thus, we analyzed phosphorylation of each representative proteins that indicated activation at 15 min and 24 h. As shown in Fig. 6, western blot analysis indicates that purified KGF-1_135_ triggered AKT (Protein kinase B) phosphorylation, which plays a key role in cellular processes such as cell proliferation, apoptosis, and cell migration. In particular, purified KGF-1_135_ more strongly activated and maintained AKT phosphorylation longer than the KGF-1_140_. ERK, a member of the mitogen-activated protein kinase, and its phosphorylated form were upregulated by treatment with KGF-1_135_ with statistically similar level to KGF-1_140_. Histone H3, which is involved in chromatin structure, was more strongly upregulated following the treatment with KGF-1_135_. After treatment of KGF-1_135_, all signaling pathways showed the trend in upregulation as that after treatment with KGF-1_140_; however, AKT and histone H3 pathways were more strongly upregulated. These results indicate that purified KGF-1_135_ stimulates the proliferation of MCF-7 cells in a manner similar to that of KGF-1_140_.

## Discussion

Growth factors that are involved in cellular processes such as proliferation, differentiation, survival, and migration via cell-to-cell communication generate interest in various applications, including cancer drugs, tissue repair, and cosmeceuticals [26, 27]. In particular, KGF-1_140_, known as palifermin, was approved by the FDA to treat severe mucositis in patients with cancer subjected to chemotherapy and radiation therapy [28, 29].

Research is underway to produce proteins on a large scale, but the production yield and protein stability remain limited. We have previously reported the effective production of growth factors using the 6HFh8 tag; however, KGF-1 was not produced in its authentic form in our process [30]. This issue is caused by the N-terminal sequence of KGF-1, which determines the cleavage efficacy of TEV protease [31]. Therefore, we selected TRX as a fusion partner and an enterokinase cleavage system that has a proteolytic activity in spite of every residue of P1′ position [32]. The fusion protein TRX-6 H-KGF-1 was expressed in a similar manner using the 6HFh8 expression system. As expected, KGF-1 was isolated from the fusion protein, but LC-MS/MS analysis and N-terminal sequencing indicated that it was a mixture of amino-terminally truncated KGF-1 variants and not full-length KGF-1 (Fig. 1d and e). For the therapeutic applications of protein, it is difficult that the mixture of two or more kinds of substances, including truncated form is approved. Therefore, it is important to produce a single protein. However, contrary to the notion that KGF-1_140_ as the most stable form [33], the more short form, KGF-1_135_ was presented in our study. Our results show that the production yield of KGF-1_135_ was improved about several dozen times compared with previously described yields: 3.1 mg/L in CHO cells with the KGF-1 isoform [34], 3 µg/mL of cell culture in *P. pastoris* [5], and 17 mg/L/OD of GST-KGF-1 fusion form and 4.8 mg/L/OD of GST-cleaved KGF-1 in *E. coli* [7].

The biological activity of full-length KGF-1 is heparin-binding properties and DNA synthesis or tyrosine kinase activity. In the cases of mutants, in vitro mutagenesis of amino-terminally truncated KGF-1 variants with up to 28 residues have heparin-binding properties [35]. Additionally, deletion of up to 10 residues has no effect on DNA synthesis or tyrosine kinase activity. However, the authors mentioned that the deletion of 29 residues reduces biological activity [35]. Currently, KGF-1_140_, which has a deletion of 23 residues is used because its clinical activity is similar to that of endogenous KGF-1, owing to its high stability [5, 33]. In the present study, KGF-1_135_, which has a deletion of 29 residues, showed biological activity similar to that of the KGF-1_140_ in MCF-7 cells (Figs. 5 and 6). The biological markers that are analyzed in this study are proliferation markers that induce DNA synthesis or tyrosine kinase activity. Thus, contrary on the previous research, the amino-terminally mutation of 29 residues maintain the biological activity. Especially, KGF-1_135_ showed higher upregulation of each signal with higher thermal stability. These results indicate that the purified KGF-1_135_ had the same effect on other KGF receptor expressing cells, such as A549 and HaCaT cells [35–38]. Our data suggested that KGF-1_135_ can be used as a drug for hair growth [8, 9] and in several clinical applications, such as wound healing [6, 39], preservation of epithelial integrity [38], DNA repair after several kinds of damage [5, 40], and colorectal or gastrointestinal disease and cancer [41, 42]. Furthermore, the high persistence of KGF-1_135_ can reduce the number of dosing so that it is possible to decrease the burden on the patient. In the present study, we designed a KGF-1 fusion protein for the simplified production of KGF-1. These simplified production processes satisfied the demand for high quality and quantity of recombinant KGF-1_135_. Our study significantly improved the stability and purification yield; however, the production yield was still low. To further enhance the production yield, advanced expression strategies such as screening for high expression levels, enhanced fusion partners, and replacing the promoter to reduce cell toxicity during induction are being considered.

## Conclusion

In this study, we discovered KGF-1_135_, the most stable amino-terminally truncated variant of KGF-1. The fusion protein TRX-6 H-D_4_K-KGF-1_135_ was successfully expressed in *E. coli* via 5 L fermentation with high solubility, indicating the possibility of massive production. KGF-1_135_ was purified (> 99%) using a two-step purification process consisting of IMAC, tag removal by hEK_L_ C112S, and cation-exchange chromatography. The characteristics and biological activity of KGF-1_135_ show that it is a possible substitute for different applications. These findings will facilitate the production of KGF-1_135_ with high quality as potential biopharmaceutical and cosmeceutical material.

## Materials and methods

### Materials

The host strain *E. coli* BL21 (DE3) (NEB, Ipswich, MA, USA) and plasmid pET-30a(+) (Merck, Darmstadt, Germany) were stored at the Korea Research Institute of Bioscience and Biotechnology (KRIBB). The full-length KGF-1 was fused to TRX via the cleavage site (DDDDK) of enterokinase, and the *E. coli*-based codon-optimized fusion gene was synthesized from DNA2.0 (ATUM, Menlo Park, CA, USA). AKTA Prime Plus, AKTA purifier system, HiTrap SP HP (5 mL), HiTrap CM HP (5 mL), HisTrap HP (5 mL), and the Superdex 75 columns were purchased from GE Healthcare Life Sciences. Restriction enzymes, DNA polymerase, the In-Fusion HD Cloning Kit, and In-Fusion HD cloning kit were purchased from Takara (Takara Bio, Otsu, Japan). KGF-1_140_ was purchased from Creative BioMart (THP-0113, NY, USA ). The antibodies phosphor-Akt (Ser473) antibody (9271), Akt Antibody (9272), Phospho-p44/42 MAPK (Erk1/2) (Thr202/Tyr204) (E10) Mouse mAb (9106), and Phospho-Histone H3 (Ser10) (D2C8) Rabbit mAb (3377) were purchased from Cell signaling Technology, Inc., Danvers, MA, USA. Anti-Erk1/2 antibody (442,675) was purchased from Calbiochem (Schwalbach, Germany). The anti-Actin antibody (A2066) was purchased from Sigma-aldrich (St. Louis, MO, USA).

### Construction and expression of KGF-1 expression vectors and strains

The synthesized TRX-6 H-KGF-1 plasmid was inserted into the pET-30a plasmid. TRX-6 H was amplified by polymerase chain reaction (PCR) using the oligopeptide primers TRX-6 H F and TRX-6 H R, GCCCTCCTTGTCGTCGTCATCACC and GGAGATATACATATGGTGAAACAGATC, respectively. KGF-1_135_ was amplified by PCR using the oligopeptide primers KGF-1_135_ F and KGF-1 135 R, GACGACAAGGAGGGCGGCGACATT and GTGGTGGTGGTGCTCGAGTTACGTAATGGCCATCGG, respectively. TRX-6 H-KGF-1_135_ was amplified by the fusion PCR, the mixture of TRX-6 H and KGF-1_135_ used for the template, using the oligopeptide primers KGF-1 135 F and TRX-6 H R. The digested and amplified products were ligated into the vector pET-30a at *EcoRI* and *XhoI* sites using In-Fusion HD cloning kit. The recombinant plasmids were transformed into *E. coli* BL21 (DE3) cells using the heat shock method as following our previous report [30]. Briefly, *E. coli* BL21 (DE3) competent cells and each plasmid were mixed and incubated on ice for 30 min. Subsequently, each mixture was subjected to heat shock at 42 °C for 40 s and incubated on ice for 2 min. Finally, curing was conducted at 37 °C for 1 h after the addition of Luria-Bertani (LB) medium. The cells were spread on an LB plate containing 50 µg/mL kanamycin and incubated at 37 °C overnight.

### Cultivation

The flask cultivation and 5 L fermentation media and method were described in our previous study [30]. Flask cultivation was performed in 1 L shake baffle flasks in 250 mL of auto-induction media containing 0.5 g/L of glucose, 3 g/L of glycerol, 2 g/L of lactose, 0.15 g/L of MgSO_4_·7H_2_O, 10 g/L of yeast extract, 16 g/L of tryptone, 3.3 g/L of (NH_4_)_2_SO_4_, 1 mL/L of trace elements, 6.8 g/L of KH_2_PO_4_, and 7.1 g/L of Na_2_HPO_4_·12H_2_O. Trace elements consisted of 0.5 g/L CoCl_2_·6H_2_O, 65 g/L FeSO_4_·7H_2_O, 3 g/L MnSO_4_·5H_2_O, 5 mL/L H_2_SO_4_ (95–98%), 0.08 g/L KI, 6 g/L CuSO_4_·5H_2_O, 20 g/L ZnCl_2_, 0.02 g/L H_3_BO_3_, 0.2 g/L Na_2_MoO_4_·2H_2_O, and 0.2 g biotin. The transformants were cultured overnight in test tubes with 3 mL of LB medium and 50 µg/mL of kanamycin; 2.5 mL of the culture was transferred into 1-L baffled flasks containing 250 mL of auto-induction medium with 50 µg/mL kanamycin and incubated on a shaker at 200 rpm and three temperature (37, 30, and 25 °C).

Fermentation (5 L) was performed using media containing 15 g/L of glucose, 1 g/L of MgSO_4_·7H_2_O, 10 g/L of yeast extract, 10 g/L of casein peptone, 10 g/L of (NH_4_)_2_SO_4_, 0.5 g/L of NaCl, 3 g/L of Na_2_HPO_4_·12H_2_O, 3 g/L of KH_2_PO_4_, 1 mL/L of trace element solution, and 50 µg/mL of kanamycin. For the additional feed solution, 600 g/L glucose and 20 g/L of yeast extract were prepared. Fermentation was controlled under the following conditions: cell growth at 37 °C, protein expression at 25 °C, pH adjusted to 7.0 with ammonium hydroxide, an airflow of 1 vvm, and agitation from 200 to 900 rpm to maintain the dissolved oxygen levels above 30%. All the controlled conditions were monitored, and glucose levels were analyzed using a glucose analyzer (YSI 2700 Biochemistry Analyzer; Yellow Springs Instrument, Yellow Springs, OH, USA). For the seed culture, a single colony of each recombinant *E. coli* from the LB plate with 50 µg/mL kanamycin was inoculated into 200 mL of the same medium in a 2 L baffled flask and incubated at 37 °C overnight. Fermentation was initiated at a working volume of 2 L. After consumption of the initial glucose, additional glucose was added at a rate of 6–7 g/L/h. When the cell density reached approximately 35, the temperature was decreased to 30 °C and glucose was added at a rate of 5 g/L/h for 0.5 h. Then, the temperature was decreased to 25 °C and the glucose feeding rate was reduced to 4 g/L/h. After 1.5 of temperature shift, lactose was added at a final concentration of 15 g/L to induce KGF-1 protein expression. After 28.5 h of fermentation, cells were harvested by centrifugation at 7,000 rpm at 4 °C for 30 min, and the harvested cells were stored at -70 °C.

To analyze the expression levels and solubility, the cells were diluted to an OD_600_ of 5. After washing twice with phosphate buffered saline (PBS), the precipitate was resuspended in the same buffer and disrupted by sonication (Cole-Parmer Instruments, Vernon Hills, IL, USA) on ice at 40% amplitude for 3s on time and 5s off time for a total of 10 min. Disrupted cells were stored for the total fraction. After removal of debris by centrifugation at 12,000 rpm at 4 °C for 5 min, the supernatant was stored for the soluble fraction. For the insoluble fraction, the debris was washed twice with PBS and resuspended in the same buffer. Protein concentration was determined using the Pierce™ BCA protein assay kit (Thermo Scientific, Waltham, MA, USA), and absorbance was measured at 550 nm using an Infinite 200 PRO plate reader (TECAN, Männedorf, Switzerland). Protein expression was analyzed by loading the protein samples and culture media onto a 4–12% Bis-Tris Plus SDS-PAGE gel (Thermo Scientific) and running at 170 V and 500 mA for 32 min, followed by staining with InstantBlue (Abcam, Cambridge, UK).

### Purification of KGF-1

For the full-length KGF-1 protein, primary purification was conducted using a 5 mL HisTrap column. The supernatant was applied to HisTrap columns at a flow rate of 3 mL/min, which were pre-equilibrated with a binding buffer (20 mM Tris-HCl pH 8.0, 300 mM NaCl). After washing with binding buffer containing 50 mM imidazole at a flow rate of 3 mL/min, TRX-KGF-1 was eluted using an elution buffer (binding buffer with 500 mM imidazole), and the eluent was dialyzed against 20 mM Tris-HCl (pH 8.0). hEK_L_ C112S (for the cleavage of the fusion protein) was purified as previously reported [32]. After cleavage with hEK_L_ C112S at 4 °C overnight, KGF-1 was purified using a HiTrap SP column. KGF-1 was applied to a HiTrap SP column at a flow rate of 3 mL/min, which was pre-equilibrated with a binding buffer (20 mM Tris-HCl, pH 8.0). After washing with a binding buffer containing 500 mM NaCl at a flow rate of 5 mL/min, KGF-1 was eluted using an elution buffer (20 mM Tris-HCl pH 8.0, 600 mM NaCl). KGF-1 concentration was measured using the BCA method, and purity was measured using C18 RP-HPLC and SDS-PAGE [30].

For the KGF-1_135_ protein, primary purification was conducted using a 5 mL HisTrap column. The supernatant was applied to the HisTrap columns at a flow rate of 3 mL/min, which were pre-equilibrated with a binding buffer (20 mM Tris-HCl pH 8.0, 300 mM NaCl). After washing with the binding buffer containing 50 mM imidazole at a flow rate of 3 mL/min, TRX-6 H-KGF-1_135_ was eluted using an elution buffer (20 mM Tris-HCl pH 8.0, 300 mM NaCl, and 500 mM imidazole), and the eluent was dialyzed against 20 mM Tris-HCl (pH 8.0). After cleavage with hEK_L_ C112S at 4 °C overnight, KGF-1_135_ was purified using a HiTrap SP column. KGF-1_135_ was applied to a HiTrap SP column at a flow rate of 3 mL/min, which was pre-equilibrated with binding buffer (20 mM Tris-HCl, pH 8.0). After washing with a binding buffer containing 500 mM NaCl at a flow rate of 5 mL/min, KGF-1_135_ was eluted with an elution buffer (20 mM Tris-HCl with 1 M NaCl). KGF-1_135_ concentration was measured using the BCA method, and purity was measured using C18 RP-HPLC and SDS-PAGE [30].

### Purity analysis of KGF-1 with HPLC

The HPLC was described in our previous study [30]. Briefly, the purity of KGF-1_135_ was evaluated using HPLC (1200 Series; Agilent Technologies, Santa Clara, CA, USA) with a UV detector at a wavelength of 214 nm. For the analysis, a C18 reverse-phase column (Zorbax Eclipse XDB, 80 Å C18, 4.6 ⋅ 150 mm, 5 μm; Agilent Technologies) was maintained at 40 °C. The column was pre-equilibrated with buffer A (0.1% trifluoroacetic acid in distilled water) and 5% (v/v) buffer B (0.1% trifluoroacetic acid in acetonitrile). The flow rate was 0.5 mL/min; the sample volume was 20 µL, and the run time for each sample was 45 min. The injected sample was separated using a gradient of 5–100% B in A for 35 min. Equilibrium was achieved after an additional 10 min.

### N-terminal sequencing and LC-MS/MS

The N-terminal sequencing and LC-MS/MS were described in our previous study [30]. Briefly, p.

rotein N-terminal sequences were obtained by transferring the purified recombinant KGF-1 and KGF-1_135_ proteins to a polyvinylidene difluoride membrane using a Procise ABI 492 protein sequencer (Applied Biosystems, Foster City, CA, USA). The authenticity of the purified proteins was verified using native mass spectrometry at eMASS (Seoul, Republic of Korea). Samples were analyzed according to the service provider’s protocol. Briefly, they were first resolved by UHPLC Ultimate 3000 (Thermo Scientific) on an ACQUITY-C8 column (2.3⋅130 mm, 1.7 μm; Waters, Milford, MA, USA). Mobile phases A [H_2_O/formic acid, 100/0.2 (v/v)] and B [acetonitrile/formic acid, 100/0.2 (v/v)] were used for the analysis. Approximately 10 µL of the sample was injected for analysis and separated using a gradient of 5–100% B in A for 12 min. Native protein mass was detected using a TripleTOF 5600+ (AB SCIEX, Framingham, MA, USA).

### CD analysis

The CD analysis was described in our previous report [43]. Far-UV CD measurements of KGF-1_135_ and KGF-1_140_ were performed using an automated Chirascan CD spectrometer (Applied Photophysics, Leatherhead, UK). Spectra were recorded over a range of 195 to 260 nm using a 0.5-mm path length at 25 °C to determine the secondary structure of KGF-1_135_. To evaluate the thermal stability of KGF-1_135_, the temperature was increased by 1.0 °C stepwise from 20 to 90 °C. The purified protein was adjusted to 0.3 mg/mL for CD analysis. The background CD spectrum of the buffer was subtracted from the CD spectrum of KGF-1_135_. Gel filtration analysis was performed using an ÄKTA purifier system with the Superdex 75 column. The KGF-1135 and protein markers were detected using a UV light detector at wavelength of 280 nm.

### Cell culture

The immortalized human breast cell line, MCF7 was maintained in Dulbecco’s modified Eagle’s medium (DMEM) supplemented with 10% heat-inactivated fetal bovine serum (FBS), 2 mmol/l glutamine, and 100 U/ml penicillin/streptomycin in 5% CO_2_ at 37 °C. Cells were then treated 20 ng/ml of the KGF-1_140_ as a control and KGF-1_135_ for 24 h.

### Immunoblot analysis

The immunoblot analysis was modified by referring to the method of the previous study [44]. After treatment under different conditions as described in the figure legends, cells were collected and lysed in M2 buffer (20 mM Tris, pH 7.6, 0.5% NP-40, 250 mM NaCl, 3 mM Ethylenediaminetetraacetic acid (EDTA), 3 mM EGTA, 2 mM dithiothreitol, 0.5 mM phenylmethylsulfonyl fluoride (PMSF), 20 mM β-glycerol phosphate, 1 mM sodium vanadate, and 1 µg/ml leupeptin). 30 µg of cell lysate was subjected to 10% SDS-PAGE and transferred to nitrocellulose membrane. After blocking with 5% skim milk in PBS/T, the membrane was probed with the relevant antibodies and visualized using Super Signal West Pico chemiluminescent Substrate kit (Thermo Fisher Scientific Inc.) according to the manufacturer′s instructions.

### Statistical analysis

All data were obtained from the independent experiments are presented as the mean ± standard deviation. The data were analyzed with Student’s t-test. Analysis of variance (ANOVA) was performed for relevant data. Values of p ≤ 0.05 were considered statistically significant, and values of p ≤ 0.01 were considered highly significant.

## Electronic supplementary material

Below is the link to the electronic supplementary material.

**Additional file 1: Table S1.** LC-ESI-MS analysis results of KGF-1.

## Data Availability

All data generated or analyzed during this study are included in this published article and its supplementary information files.

## Abbreviations

GST: glutathione-S-transferase
hEK_L_ C112S: human enterokinase light chain C112S
IMAC: immobilized metal affinity chromatography
KGF-1: keratinocyte growth factor 1
TRX: thioredoxin
