# Supplementary material for: Production of a 135-residue long N-truncated human keratinocyte growth factor 1 in Escherichia coli
Source: Microb Cell Fact. 2023 May 11;22:98. doi: 10.1186/s12934-023-02097-z (PMC10173505; doi:10.1186/s12934-023-02097-z)
Supplement: Supplementary file 1 — Additional file 1: Table S1. LC-ESI-MS analysis results of KGF-1. [file 12934_2023_2097_MOESM1_ESM.docx]

**Table S1.** MS analysis results of KGF-1 by LC-ESI-MS. The red color indicated the mass peak variant of KGF-1 and the pear number of remarks indicated the majority of each fragment.

| **Mass** | **Area** | **Height** | **Width** | **Width at 50%** | **Resolution** | **Charge** | **Monoisotopic** | **Mass (charge)** | **Mass/charge (charge)** | **Remarks** |
| --- | --- | --- | --- | --- | --- | --- | --- | --- | --- | --- |
| 15617.80467 | 213454.0392 | 27072.86516 | 22 | 7.034774419 | 2220.086067 | 0 | Yes | *15616.8 | *15617.8 | Peak 1 |
| 15633.67261 | 23721.18301 | 2835.727334 | 12 | 9.422211363 | 1659.236033 | 0 | Yes | *15632.7 | *15633.7 |  |
| 15649.74293 | 43214.43453 | 4799.730682 | 19 | 7.989817537 | 1958.710929 | 0 | Yes | *15648.7 | *15649.7 |  |
| 15911.37037 | 33910.55252 | 4114.455016 | 40 | 7.901504756 | 2013.713952 | 0 | Yes | *15910.4 | *15911.4 |  |
| 16027.28602 | 269939.8521 | 31683.93574 | 22 | 8.115672395 | 1974.856209 | 0 | Yes | *16026.3 | *16027.3 | Peak 2 |
| 16043.74283 | 37080.06429 | 4521.060649 | 11 | 9.047600701 | 1773.259382 | 0 | Yes | *16042.7 | *16043.7 |  |
| 16059.33354 | 89358.78192 | 9820.918002 | 19 | 8.254401289 | 1945.547954 | 0 | Yes | *16058.3 | *16059.3 |  |
| 16091.1567 | 16659.70575 | 1841.914422 | 18 | 7.83215489 | 2054.499294 | 0 | Yes | *16090.1 | *16091.2 |  |
| 16277.53796 | 284475.0073 | 31866.5382 | 21 | 8.447634022 | 1926.875373 | 0 | Yes | *16276.5 | *16277.5 | Peak 3 |
| 16293.30946 | 56904.76161 | 6858.313285 | 12 | 8.849777289 | 1841.098247 | 0 | Yes | *16292.3 | *16293.3 |  |
| 16309.52605 | 74338.27523 | 7336.476049 | 19 | 9.003693949 | 1811.426082 | 0 | Yes | *16308.5 | *16309.5 |  |
| 16325.35851 | 18631.09182 | 1958.338058 | 13 | 10.2002687 | 1600.483183 | 0 | Yes | *16324.4 | *16325.4 |  |
| 16340.99987 | 18107.90715 | 1901.661426 | 17 | 9.412921604 | 1736.017845 | 0 | Yes | *16340.0 | *16341.0 |  |
